# Assessing the performance of Portuguese public hospitals before and during COVID-19 outbreak, with optimistic and pessimistic benchmarking approaches

**DOI:** 10.1007/s10729-024-09693-4

**Published:** 2024-11-28

**Authors:** Guilherme Mendes Vara, Marta Castilho Gomes, Diogo Cunha Ferreira

**Affiliations:** 1 CEGIST, Instituto Superior Técnico, Universidade de Lisboa, Av. Rovisco Pais, 1, 1049-001 Lisbon, Portugal; 2 CERIS, Instituto Superior Técnico, Universidade de Lisboa, Av. Rovisco Pais, 1, 1049-001 Lisbon, Portugal; 3 Centre for Public Administration and Public Policies, Institute of Social and Political Sciences, Universidade de Lisboa, Rua Almerindo Lessa, 1300-663 Lisbon, Portugal

**Keywords:** Healthcare performance, Hospital efficiency, COVID-19 pandemic, Composite indicators, Benefit-of-the-Doubt, Benchmarking

## Abstract

**Abstract:**

The COVID-19 pandemic had a profound impact on the tertiary sector, particularly in healthcare, which faced unprecedented demand despite the existence of limited resources, such as hospital beds, staffing resources, and funding. The magnitude and global scale of this crisis provide a compelling incentive to thoroughly analyse its effects. This study aims to identify best practices within the Portuguese national healthcare service, with the goal of improving preparedness for future crises and informing policy decisions. Using a Benefit-of-the-Doubt (BoD) approach, this research constructs composite indicators to assess the pandemic's impact on the Portuguese public hospitals. The study analyzes monthly data from 2017 to May 2022, highlighting critical trends and performance fluctuations during this period. The findings reveal that each COVID-19 wave led to a decline in hospital performance, with the first wave being the most severe due to a lack of preparedness. Furthermore, the pandemic worsened the disparities among examined hospitals. Pre-pandemic top performers in each group improved their performance and were more consistently recognized as benchmarks, with their average benchmark frequency increasing from 66.5% to 83.5%. These top entities demonstrated greater resilience and adaptability, further distancing themselves from underperforming hospitals, which saw declines in both performance scores and benchmark frequency, widening the performance gap. The superior performance of top entities can be attributed to pre-existing strategic tools and contextual factors that enabled them to withstand the pandemic's challenges more effectively.

**Highlights:**

• The pandemic aggravated the differences between the hospitals examined.

• The top-performing entities further distanced themselves from the remaining entities after the pandemic

• Entities considered benchmarks before the pandemic remained the same, and became even more consistent during the pandemic.

• The top-performing entities achieved higher scores than their pre-pandemic performance levels.

• Benchmarking models for composite indicators with diverse decision-making preferences, and treatment of imperfect knowledge of data.

## Introduction

In the last quarter of 2019, a previously unknown disease emerged in Wuhan, China, being later identified as COVID-19 [1]. This virus soon became a worldwide problem, causing widespread disruption, illness, and suffering. The disease was declared a global pandemic by the World Health Organization (WHO) in March 2020 [2], but the world is yet to recover from the shock and repercussions. The rapid propagation along with the severity of the cases (and lack of treatment) forced governments to impose strict regulations to prevent an unmanageable virus dissemination. Such regulations, although necessary, led to unparalleled consequences for the global economy, across all sectors [3].

The healthcare sector was one of the most stressed, having to ensure basic health services while having workforce shortages, limited medical supplies, and an infrastructure not capable of handling the unprecedented demand during every COVID-19 wave [4]. Still suffering from the repercussions of the 2008 financial crises, the healthcare sector continues to be underfunded in many countries, particularly in the southern European ones, where the heavy austerity forced major budgetary cuts [5]. Additionally, the problems in this sector are not solved by “throwing money” at them, that will only worsen the inefficiencies [6]. Instead, policies and changes must be implemented over time with clear intentions on how to use the available resources [7].

In Portugal, spending on the health sector has consistently been among the highest annual expenditures, totalling about 23.7 billion euros in 2021 and accounting for 11% of the gross domestic product (GDP) for that year (INE, 2022).^1^ Notably, public hospitals alone contribute significantly to this expenditure, accounting for 7.5 billion euros, highlighting the need for these healthcare institutions to experience profound changes to minimize waste [8]. On top of that, COVID-19 eventually took over the Portuguese budgetary planning, controlling the pace of the financial system, with almost no margin for failure [9]. During this period, statistics were crucial in countries’ efforts to combat the virus, particularly when deciding on the type and degree of budgetary constraints.

While the significant economic and financial costs of the National Health Service (NHS) during the pandemic may dissuade the decision makers from spending additional public funds, consistent investment should be the main focus not only to upgrade the current facilities and workforce, but also to achieve more efficient practices [10]. Decision-makers must be proactive rather than reactive through continuous changes and measures [11].

Several studies have been applied to assess the performance of public health services, with a predominant focus on employing non-parametric frontier methodologies for that purpose [12–15]. For determining a frontier, the best known and most widely used methodology is the Data Envelopment Analysis (DEA) [16]. Remarkably, the Portuguese case study has had a significant impact on the literature, as evidenced by its widespread publication and relevance to social issues [17–19].

As the healthcare sector returns to pre-pandemic levels, analysing the extensive and readily available data from public Portuguese hospitals before and during COVID-19 can offer valuable insights for future healthcare improvements. In this study, we use the Benefit-of-the-Doubt (BoD) approach [20], a specific type of DEA methodology, to combine key performance indicators (KPIs) related to access, clinical safety, productivity, and care appropriateness, into a single performance score. These KPIs are essential to the Portuguese government for evaluating healthcare performance. Composite Indicators (CIs) are valuable here because they create a metric that represents each entity's performance and how it changes over the study period.

Overall, this work has the following objectives and respective research questions: (i) Assess the pandemic's impact—Did the COVID-19 pandemic have an impact on the performance of Portuguese public hospitals, and to what extent?; (ii) Analyse benchmark consistency—Are the Portuguese public hospitals considered as benchmarks the same before and during the pandemic?; (iii) Assess best practices—Was the impact equally widespread or were there entities better prepared and more resilient to the challenges brought by the COVID-19 crisis brought?

In essence, the fundamental innovations of this paper are: (i) It assesses both optimistic and pessimistic perspectives, granting a robust analysis of the actual impact of the pandemic on the Portuguese public hospitals; (ii) It introduces and explores the effects of different types of restrictions on the methodology and their impact on the CI score; (iii) It utilizes monthly data, instead of yearly data, which allows for a more detailed understanding and preview of the changes in performance and their evolution; (iv) It builds a single CI, whose meaning goes beyond efficiency to encompass other dimensions related to quality; (v) It has an extended timeframe comparing pre-pandemic performance with data until May 2022.

The subsequent sections of this study are structured as follows. Section 2 provides a representation of the literature focusing on the methods employed; however, an extensive review goes beyond the scope of this paper. Section 3 details the methodology used and the main reasons for its choice. Section 4 presents the description of the case study, and the data collected. Section 5 displays the results and potential implications. Section 6 outlines the key conclusions, recommendations, limitations and avenues for future research and potential enhancements of this study.

## Literature review

### Healthcare and the impact of the COVID-19 pandemic

Before the pandemic, healthcare services would focus on containing costs, stretching as much as possible the number of patients per worker [21]. However, the pandemic has highlighted the importance of balancing efficiency with preparedness and capacity building [22]. As healthcare services were pushed to their limits during the pandemic, there has been a greater recognition of the need for investing in robust healthcare infrastructure, including adequate staffing levels, medical equipment, and supplies, in order to be able to respond effectively to unexpected crises [23, 24].

Many researchers have conducted studies on the effects of the pandemic on healthcare services and have shown that COVID-19 has led to increased demand for them [25]. The pandemic has also highlighted existing healthcare disparities and the need for healthcare services to be more resilient and prepared for future crises [7]. Table 4 (see Appendix) gives a detailed overview of works about the impact of the COVID-19 pandemic and its effects on healthcare. Based on our own perception, three distinct streams were identified in the literature on the effects of the pandemic:

- The impact of COVID-19 on healthcare workers [26, 27]. These reviews have identified challenges such as burnout, anxiety, and stress among healthcare workers, and highlight the need for better support and resources for frontline healthcare workers.
- The impact of COVID-19 on care and efficiency [9, 28–30]. The authors identified challenges, including delays in diagnosis and treatment, reduced access to screening, and changes in treatment protocols.
- The impact of COVID-19 on mental health services [31–33]. These services have seen increased demand, but people have also been hesitant to seek help due to fears related to the pandemic.

This study primarily examines the impact of COVID-19 on the performance and efficiency of healthcare management units. Although prior research, such as Kamel et al. [34] and Nepomuceno et al. [35], has touched upon this subject, their main emphasis has often been on productivity and efficiency. There has been a tendency to overlook crucial elements like access, clinical safety, and care appropriateness, as highlighted by Ferreira et al. [36]. However, a limited amount of literature, like Henriques & Gouveia [9] and Caldas & Varela [37], has explored these additional dimensions which extend beyond efficiency. Nonetheless, these studies have explored these dimensions separately, while in this study the dimensions are all integrated into a single CI, rather than evaluating them separately.

Another distinctive characteristic of this study is its extended timeframe. Unlike many studies that primarily centre on the pandemic's early stages, this analysis spans more than 3 years to establish a baseline and is extended beyond the initial year of the pandemic, covering the following 26 months. This perspective provides insights into whether top performers before the pandemic maintained their excellence during this challenging period and whether their pre-pandemic performance influenced how they were affected by the pandemic.

### Data envelopment analysis in the Portuguese healthcare system

When it was necessary to assess performance and make changes to the usage of resources, the traditional approaches were either a regression analysis or an application of performance indicators [38]. However, since the beginning of the millennium, DEA gained traction as a general method for benchmarking and efficiency measurement, especially in healthcare [39–42].

The most commonly used input and output variables in healthcare DEA studies include bed occupancy rate, staffing levels, length of stay, number of patient visits, and operating expenses [41, 43, 44]. These variables are used to identify the most efficient healthcare providers and to analyse the impact of healthcare policies on efficiency [45]. In this study, the KPIs employed are constructed based on these frequently used variables, such as bed occupancy rate and rate of appointments within a specified timeframe [18, 46–48].

Overall, there is evidence to suggest that efficiency in the Portuguese healthcare sector has improved over time [49], although potential for significant improvements in resource utilization and patient outcomes through better management and policy interventions remain apparent. Additionally, as outlined by Kohl et al. [41], it is crucial to bridge the gap between the academic application of DEA in healthcare and its practical relevance for policymakers, economists, and healthcare managers. To accomplish this, two key approaches have been suggested: firstly, the development of methods to demonstrate the reliability of DEA models, and secondly, closer collaboration with hospital managers to validate results and report successful efficiency improvements.

Table 5 (see Appendix) presents a synopsis of chosen papers concerning the DEA approach in the healthcare sector. These papers were chosen because they concentrate on the Portuguese context, except for Kohl et al. [41], which covers a review of studies where DEA was applied to the healthcare sector.

### Linear benefit-of-the-doubt for constructing composite indicators

Constructing CIs is a complex task that involves selecting and weighting individual indicators based on their significance to the overall concept being measured and combining them into a single score. In healthcare management, CIs can be used to monitor and evaluate various aspects of healthcare delivery, including efficiency, effectiveness, patient satisfaction, and organizational performance [50]. They help healthcare managers and policymakers make informed decisions by providing a consolidated and easily interpretable summary of complex data and trends.

However, the subjective nature of CIs often poses challenges. The BoD approach emerges as a potential solution, leveraging linear optimization to derive weighting schemes directly from the data, rather than relying on the opinions and preferences of Decision making units (DMUs). Since Cherchye et al. [20] proposed the BoD approach, several studies have expanded upon the BoD approach, addressing its limitations through various developments and integrations, such as:

- Construction of a worst-case scenario rather than an ideal scenario for the pessimistic approach [51]. In essence, it provides a more conservative viewpoint, offering valuable insights into the potential limitations or vulnerabilities of DMUs. This is particularly relevant in contexts where risk mitigation and robust decision-making are paramount. By considering the worst possible scenarios, it enables analysts to identify critical areas for improvement and assess the resilience of DMUs under adverse conditions.
- Combination between BoD and Multi-Criteria Decision Analysis to find a set of shared weights for all DMUs [52]. This integration develops a more comprehensive and informed decision-making process where decision-makers can weigh the relative importance of different criteria, account for uncertainties, and assess the robustness of their choices.
- Inclusion of directional distance measures to allow KPIs to expand at different rates [53, 54]. This flexibility is valuable when dealing with complex scenarios where some metrics may naturally grow or change at different rates over time.
- Enhancing the explanatory power by incorporating non-discretionary variables/external factors [55]. Through this, it is recognized that DMUs often operate in contexts influenced by external conditions, which enriches the approach by accounting for factors beyond managerial control.
- Increasing the accuracy and reliability of the approach through the mitigation of the impact of outliers and extreme values [55]. Outliers can significantly distort the efficiency scores for the entire dataset by pushing the efficient frontier far away from the majority of the data points.
- Incorporating and treating undesirable (or reverse) KPIs [54, 56]. This ensures that the BoD approach can effectively accommodate KPIs where lower values indicate superior performance, alongside those where higher scores denote better performance. This modification allows for the inclusion of KPIs that were previously challenging to incorporate due to their reverse nature.
- Fixing minimum and maximum thresholds for the entity to be considered a benchmark [17]. This innovation addresses a critical issue in benchmarking, providing clear criteria for identifying benchmark entities. Such thresholds ensure that only relevant entities are considered benchmarks.

This study, while incorporating some of these advancements, also aims to investigate the influence of different weight restrictions on the final performance score. Table 5 (see Appendix) offers a detailed description of the above advancements made on the linear BoD approach.

## Methodology

### Data envelopment analysis and the benefit-of-the-doubt approach

Charnes et al. [16] introduced the DEA approach as a tool for use in public sector and not-for-profit institutions, where the presence of several inputs (resources) and outputs (products, services) makes evaluation difficult. The model, called Charnes, Cooper, and Rhodes Model (CCR), assumes a fictional frontier of efficient DMUs made up of a weighted average between existing entities, without the need for the a priori specification of weights. One of the main limitations is that the authors assume that all DMUs operate at the same scale and that scale is optimal, with constant returns to scale.

Based on the work above, Banker et al. [57] proposed a model, called Banker, Charnes, and Cooper Model (BCC), introducing a new variable to distinguish whether operations were conducted in regions of decreasing, constant, or increasing returns to scale, allowing for varying scale levels. The difference between this work’s frontier, and Charnes et al. [16] frontier, is the scale efficiency.

The BoD approach, rooted in DEA, was introduced by Melyn & Moesen [58] to assess macroeconomic performance and has since been adapted for constructing composite indicators (CI). Cherchye et al. [20] extended the BoD approach to deal with cases where there are multiple reference units, resulting in performance scores that offer a broader interpretation beyond just efficiency. This approach can be understood as a DEA model with variable returns to scale, output-oriented, and utilizing unitary inputs [17]. Essentially, this approach involves giving the DMU the ‘benefit of the doubt’ and assuming that it is operating as efficiently as possible unless clear evidence suggests otherwise. The underlying idea is that a DMU may actually be more efficient than it appears based solely on the available data.

The novelty of this study's approach lies in two key contributions. First, the introduction of new constraints into the existing framework. These constraints aim to explore the degree of non-compensability they grant to the methodology, by limiting the degree to which inefficiencies in certain inputs can be offset by others. Second, the possibility of performing statistical inference on the efficiency distribution of each entity, through imputation, rather than relying on static efficiency scores. This shift opens the door to deeper insights, allowing for hypothesis testing and the construction of confidence intervals around efficiency estimates [59].

### Optimistic benefit-of-the-doubt with multipliers’ weight constraints

In this approach, sub-indicators are linearly aggregated into one single index in the ‘multiplier’ arrangement, as stated by Cherchye et al. [20]. By construction, the index has the lower limit of 0 and upper limit of 1 (best result, meaning that the entity is a benchmark). The model is as follows:

1 $${CI}_{t}^{opti}=\underset{{\alpha }_{t,j}}{\text{max}}\sum_{j=1}^{n}{\alpha }_{t,j}\bullet {y}_{t,j}$$

2 $$s.t.\sum_{j=1}^{n}{\alpha }_{t,j}\bullet {y}_{i,j}\le 1 ,m\text{ constraints},\text{ one for each entity }i$$

3 $$\alpha_{t,j}\geq0,\;n\;\mathrm{constraints},\;\mathrm{one}\;\mathrm{for}\;\mathrm{each}\;\mathrm{variable}\;j$$

where ${CI}_{t}^{opti}$ is the composite indicator for entity $t (i=1,\dots ,t,\dots ,m)$; ${y}_{t,j}$ is the value for the entity $t$ on the variable $j (j=1,\dots , n)$; ${\alpha }_{t,j}$ is the multiplier associated to the value of the variable $j$ of the entity $t$. Equation (1) is the objective function, where the most favourable multipliers are chosen to achieve the maximum ${\text{CI}}_{\text{t}}$ possible, while respecting the constraints. Equation (2) ensures that the maximum achievable score is one. Equation (3) guarantees that the multipliers are nonnegative.

Assigning appropriate weights to variables is crucial for accurate efficiency measurement as it reflects the relative importance of different variables in the production process. Without appropriate weights, the efficiency scores can be misleading and may not reflect the true score of a unit [60]. Through this, the incorporation of weight restrictions allows for the DMUs to weigh in their preferences while still leaving the process objective [20]. For this, a last condition is added to the previous model, arriving at the best possible score for each entity:

4 $$\frac{{\alpha }_{t,j}}{\sum_{i=1}^{n}{\alpha }_{t,i}} \ge {w}_{j} ,n\text{ constraints},\text{ one for each variable }j$$

Equation (4), is a multiplier weight restriction, which ensures that every multiplier has a weight of at least ${w}_{j}$ in relation to the sum of all the multipliers for the variable $j$.

### Pessimistic benefit-of-the-doubt with multipliers’ weight constraints

Unlike the model in Sect. 3.2, the pessimistic approach allocates lower weights to variables where the entity performs well and higher weights to variables where the entity performs worse, thus arriving to the worst possible score. The formulation is now a minimization problem and is as follows:

5 $${CI}_{t}^{pessi}=\underset{{\alpha }_{t,j}}{\text{min}}\sum_{j=1}^{n}{\alpha }_{t,j}\bullet {y}_{t,j}$$

6 $$s.t.\sum_{j=1}^{n}{\alpha }_{t,j}\bullet {y}_{i,j}\ge 1 ,m\text{ constraints},\text{ one for each entity }i$$

7 $$\alpha_{t,j}\geq0,\;n\;\mathrm{constraints},\;\mathrm{one}\;\mathrm{for}\;\mathrm{each}\;\mathrm{variable}\;j$$

8 $$\frac{{\alpha }_{t,j}}{\sum_{j=1}^{n}{\alpha }_{t,j}} \ge {w}_{j} ,n\text{ constraints},\text{ one for each variable }j$$

where the objective is to minimize Eq. (5), and the restrictions in Eq. (6) must be equal or greater than one, rather than equal or less. Equations (7) and (8), guarantee the nonnegativity and ensure they satisfy the weight restrictions, respectively.

However, the score this model provides is not comparable to the optimistic score, as it measures how close each entity is to the “frontier of worst cases”. As a result, a linear scaling in the min–max range must be done to allow comparison as stated ahead, in Sect. 3.5.

Lastly, as the number of variables increases, a notable trend emerges: the number of efficient entities tends to increase correspondingly [61]. In scenarios where multiple entities are tied in terms of their performance under the best-case scenario, the worst-case scenario analysis helps to further rank and differentiate these tied entities [51]. By considering the worst-case scenario, the study assesses the entities based on the most unfavorable conditions evaluating their potential drawbacks and vulnerabilities [62].

### Optimistic and pessimistic benefit-of-the-doubt with pie-share weight constraints

This section introduces a version of the BoD model that incorporates proportional variable share constraints. This approach ensures that each variable receives a minimum share in the final score, rather than each multiplier, regardless of the performance of each entity on that variable. This provides a normalization to the scale of each variable, ensuring a fair evaluation of entities by adjusting the values to a common scale. In other words, this enhancement addresses a potential issue with the previous weight restrictions, Eqs. (4) and (8), which only imposed limitations on the multipliers, rather than the variables. This formulation is similar to the initial model by Cherchye et al. [20] in Sect. 3.2, with the addition of the new restriction to the weights:

9 $$\frac{{\alpha }_{t,j}\bullet {y}_{t,j}}{\sum_{j=1}^{n}{\alpha }_{t,j}\bullet {y}_{t,j}} \ge {w}_{j} ,n\text{ constraints},\text{ one for each variable }j$$

this restriction is applied to both optimistic and pessimistic approaches and the key difference between the earlier restrictions, described in Eqs. ( 4 ) and ( 8 ), and Eq. ( 9 ) is that the former impose a minimum value solely on the weights of the multipliers. In constrast, Eq. ( 9 ) requires each variable to have a minimum share of the total value, ensuring that every variable contributes a certain minimum proportion to the overall total.

This new constraint imposes a greater punishment on entities with very poor performance on at least one variable. This is because if an entity has a very low value for a given variable, this restriction ensures that this variable is not undervalued in the final score, as it would be in the models with the multipliers’ weight constraints. Furthermore, this approach makes it easier for experts to express their opinion on the importance of each variable, rather than on the importance of each multiplier [63]. This arises from the fact that variables are more easily understood and quantified than multipliers, which can be more abstract and difficult to interpret.

### Linear scaling between models

The following linear scaling formula was used to allow the comparison of the optimistic and pessimistic values of the BoD. The formula scales the range of the BoD values to a common scale, allowing for a direct comparison between both models.

10 $${CIfinal}_{t}^{pessi}=\frac{{CI}_{t}^{pessi}-min({CI}^{pessi})}{\mathit{max}\left({CI}^{pessi}\right)-min({CI}^{pessi})}$$

where ${CIfinal}_{t}^{pessi}$ represents the pessimistic score after transformation, on a scale from 0 to 1, where 1 signifies the maximum performance and 0 indicates the lowest possible performance value.

## Case study: Portuguese NHS

Established in 1979, the Portuguese National Health Service (NHS) has undergone notable reforms, including a late 1990s regionalization model to decentralize decision-making and better address local needs, which have improved efficiency and accessibility [64]. However, persistent challenges such as staff shortages and funding issues, compounded by an aging population and economic downturns, continue to pose significant obstacles [19]. Public–private partnerships have been introduced to improve efficiency and resource management [65]. These partnerships allow private entities to manage public hospitals while maintaining government oversight, aiming to alleviate pressure on the NHS. With the recent economic recovery, further governance reforms have been proposed to reinforce access, improve service quality, and increase efficiency [8].

### Portuguese public hospitals

Health care services are provided, in each region, by Hospitals and Health Centres. In reality, Health Centres have a weakened position in relation to the Hospital [66], partially due to their lack of autonomy. This is despite the fact that, at a political level, primary health care has always been considered the basis of the Health System and, therefore, a political priority [64]. This gap between reality and actual practice is visible in the distribution of funds, equipment and human resources [67]. As a result of this discrepancy, there is a supply of public outpatient services that cannot meet the needs of the population. This leads to an increase in the number of cases in hospital emergency services [68, 69], which raises problems at two levels. On one side, the functioning of hospital services is seriously undermined by an excessive use of emergency services, and on the other, it may cause serious problems of accessibility to primary health care. Additionally, there is an uneven regional distribution of primary care services, with a clear bias towards the Littoral districts, which reinforces the lack of equity in access to health [70].

To worsen the already stressed hospital emergency services, the COVID-19 pandemic made public hospitals the forefront of the response. They had to significantly increase their capacity to treat patients with the virus. This has included the use of temporary hospitals, the expansion of intensive care units, the deployment of additional healthcare professionals, and the resilience of healthcare workers [48].

Nowadays, with the pandemic behind us, services are back to normal levels [30]. Nevertheless, the hole left in the financial sheets of the public funds led to an increase in debt and has affected the financial performance of the health sector as a whole [71].

### Entities and variables characterization

The data used in this study were extracted from the Central Administration of the Health System (ACSS) official website,^2^ which is a Public Institute, integrated in the State's indirect administration, with administrative and financial autonomy, which executes the orientations of the Ministry of Health and is under its superintendence and tutelage.

The analysis included a total of 40 entities, as listed in Table 7 (see Appendix). Currently, ACSS has divided public hospitals into five groups, denoted from group B to group F (group A no longer exists), with the latter reserved for oncology centres and not included in this study to ensure homogeneity, allowing for an impartial comparison [72]. The hospitals groups were classified by ACSS using hierarchical clustering after standardising the variables with the capacity to explain provider costs through principal component analysis [73]. Consequently, the groups were organized based on provider costs, and are labelled in ascending order, with group B having the lowest costs and group E having the highest [74]. This labelling reflects the volume and complexity of the healthcare services provided which, in turn, determines their corresponding funding. In this study, separate BoD models were run for each ACSS group, resulting in a different frontier for each. This approach helps to avoid bias in the results due to the different operating characteristics of each group and ensures that each entity is evaluated based on its relevant peer group.

The selection of output variables was made after conducting a literature review, taking into consideration their relevance, quality, and availability [18, 46–48, 75]. Importantly, these chosen variables align with the priorities of the Ministry of Health and are a part of the annual contracts between the hospitals’ management and the ministerial tutelage. Consequently, they are deemed highly relevant to the primary stakeholder [64]. These variables are categorized into four groups, as per the ACSS: access, clinical safety, productivity, and care appropriateness.

- Access—ease and timeliness with which individuals can access healthcare services (e.g., waiting times and service availability).
- Clinical safety—extent to which healthcare services are delivered without harm or adverse events to patients (e.g., errors, infections, and other risks associated with medical treatment).
- Productivity—how effectively healthcare providers utilize their resources (e.g., staff, equipment, funds) to deliver care and achieve desired health outcomes.
- Care appropriateness—suitability and effectiveness of healthcare interventions in meeting the needs of patients.

Table 1 presents the 16 variables that were utilized in this case study, along with their desired direction. For desirable variables, an increase in value is positive, while for undesirable variables, a decrease in value is positive. Moreover, one of the variables, inpatient bed occupancy rate, is considered neutral, and its optimal value lies between 80 and 90%, with a linear decrease when below 80% or above 90%. The direction of these variables was established through data analysis and examination of graphs provided by the ACSS on its official website. Descriptive statistics for the variables are also included in that aforementioned table.

**Table 1:** List of considered variables: description, descriptive statistics, and the desired direction of change for the considered variables

Dimension	Variables	Description	Direc^1^	Mean	SD	Min	Max
**Access**	(V1) Rate of first medical appointments within time	First medical appointments within the maximum legal time of response for a first appointment in hospitals divided by the first medical appointments in percentage	**↑**	70.7%	14.5%	3.40%	100%
	(V2) Rate of patients enrolled in surgical list within time	Patients enrolled in surgical list within time divided by the total number of patients enrolled in the surgical list in percentage	**↑**	72.6%	15.4%	25.41%	100%
**Clinical safety**	(V3) Pressure ulcer rate per 1,000	Episodes with pressure ulcers divided by the number of episodes with exclusions for episodes with ulcers per 1000 inpatients	**↓**	0.8‰	2.0‰	0‰	25.2‰
	(V4) Central venous catheter-related bloodstream infections per 1,000	Central venous catheter-related bloodstream infections episodes divided by the number of episodes with exclusions for bloodstream infections episodes per thousand inpatients	**↓**	0.2‰	0.6‰	0‰	7.4‰
	(V5) Postoperative pulmonary embolism/deep vein thrombosis per 100,000	Postoperative pulmonary embolism or deep vein thrombosis episodes divided by the number of postoperative pulmonary embolism or deep vein thrombosis exclusion episodes per 100,000 surgical procedures	**↓**	236.7	345.1	0	4095.6
	(V6) Postoperative septicemia rate per 100,000	Episodes with postoperative sepsis divided by the number of episodes with exclusions for episodes with postoperative sepsis per 100,000 surgical procedures	**↓**	644.9	949.9	0	12500
	(V7) Rate of instrumental vaginal deliveries with severe lacerations	Episodes of obstetric trauma during instrumental vaginal delivery divided by the total number of episodes with exclusions for the case	**↓**	2.4%	4.4%	0%	100%
	(V8) Rate of non-instrumented vaginal deliveries with severe lacerations	Episodes of obstetric trauma during non-instrumental vaginal delivery divided by the total number of episodes with exclusions for the case	**↓**	0.5%	1.1%	0%	16.7%
**Productivity**	(V9) Standard patients per FTE doctor	Standard patients^**2**^ divided by the Full-time Equivalent (FTE) doctors	**↑**	5.7	1.5	0	16.9
	(V10) Standard patients per FTE nurse	Standard patients divided by the FTE nurses	**↑**	3.6	1.2	0	9.5
	(V11) Inpatient bed occupancy rate	Acute admission days divided by the number of acute beds multiplied by 30.4375^**3**^ and by the accumulated number of months	**○**	85.1%	46.5%	0%	1867%
	(V12) Waiting time before surgery	Number of days until surgery in homogeneous diagnostic groups scheduled inpatient surgical episodes divided by total number of episodes for this case	**↓**	0.9	0.4	0.03	4.5
**Care Appropriateness**	(V13) Rate of outpatient surgeries on potential outpatient procedures	Outpatient surgical episodes with outpatient procedures divided by the number of inpatient and outpatient surgical episodes with outpatient procedures in percentage	**↑**	82.3%	11.6%	0%	100%
	(V14) Rate of readmissions within 30 days after discharge	Readmissions within 30 days of discharge divided by the total number of admissions with discharge in the period in percentage	**↓**	7.4%	1.9%	0%	14.8%
	(V15) Rate of inpatients staying more than 30 days	Admissions with stay of over 30 days divided by the total number of admissions with discharge in the period in percentage	**↓**	3.9%	1.6%	0%	37.9%
	(V16) Rate of hip fracture surgery in the first 48 h	Patient episodes aged > = 65 years, with main diagnosis 820, with surgery performed within the first 48 h after admission divided by the total number of surgery performed for this case	**↑**	47.0%	24.2%	0%	100%

Direction^1^: ↓ The lower, the better (undesirable); ↑ The higher, the better (desirable); ◯ Neutral, optimal value between 80 and 90%

Standard patients^2^ → Hypothetical patient with a specific set of medical conditions and characteristics that are used to standardize the measurement and allow comparison of healthcare outcomes

30.4375^3^ → Number of days in each month, 365.25/12 = 30.4375

### Methodological considerations in relation to sample characteristics and data handling

By employing monthly data, it becomes possible to conduct a more comprehensive analysis of the changes, allowing a better understanding of the impact of the pandemic. This is because monthly data provides more granular information about the fluctuations and trends in the data over time, which cannot be captured by yearly data alone. These include seasonal variations, short-term fluctuations, or sudden changes in the data. To conduct a thorough analysis, the initial step involved aggregating the data into time periods of three and six months to ensure there were no frontier shifts when comparing the results to those obtained with monthly analysis. Importantly, this window analysis revealed a stable performance landscape, with no significant frontier shifts over the study period. As a result, a meta-frontier analysis was conducted, allowing to cover the entire study period with monthly data and ensuring the robustness of the analysis by comparing the best-performing entities within their respective peer groups throughout the entire period.

The data were gathered from January 2017 to May 2022, providing a robust foundation for pre-pandemic analysis and capturing the entire influence of the pandemic. The sample consists of 40 hospitals over 65 months, resulting in 2600 unique entities. With 16 variables for each entry, the total number of entries is 41,600.

The BoD approach does not take into consideration undesirable variables (variables where lower values are better, such as the waiting time), unless the model is modified accordingly [54, 56]. To address this issue, the data were standardized into an ascending scale from 0.01 to 1, using Eq. ( 11 ). The lower bound could not be zero to avoid conflicts with the weight restrictions which are undefined for null values. However, this transformation results in dimensionless multipliers, losing interpretation power.

11 $${y}_{i,j}=\frac{0.99\bullet ({y}_{i,j}^{unstd}-\text{min}({y}_{j}^{unstd}))}{\mathit{max}\left({y}_{j}^{unstd}\right)-min({y}_{j}^{unstd})}+0.01$$

where ${y}_{i,j}$ is the final standardized value of variable $j$ of entity $i$; ${y}_{i,j}^{unstd}$ is the raw data, unstandardized; and ${y}_{j}^{unstd}$ is the vector of values for the variable $j$ from all the entities.

Due to the characteristics of DEA, and benchmarking models in general, these methods are especially sensitive to the condition of the data, which can drastically shift the results and entity performance classification [76]. Imperfect knowledge of data can vary from blank entries to wrong values, where missing information is perhaps the most common problem. A more detailed discussion on imperfect knowledge of data can be found in Ferreira et al. [17], Ebrahimi et al. [77] and Wang et al. [78].

In this study, the missing data observed can stem from a variety of factors, including data collection errors, incomplete reporting, and technical issues during data transmission. Additionally, it's important to note that some data may not have been made available online yet, as it undergoes validation by the central authority before being published or made publicly accessible. To address this issue, rather than removing entities or replacing missing data with averages, multiple imputation was employed, following a similar approach to Ferreira et al. [17]. For each missing value (approximately 3% of the total sample), 5000 values were randomly generated from a normal distribution within a fixed interval, defined by the minimum and maximum values of each variable. By carrying out this, no potentially valuable benchmarks are excluded, and the data is not manipulated. All possibilities are taken into consideration and instead of working with fixed performance values, each entity is assigned a statistical distribution of performance in each month.

Lastly, the value for ${w}_{j}$ in every model was set to 0.03125, as there was no opportunity and availability for an expert’s opinion on the matter. This value was obtained by dividing one by the product of twice the number of variables ($16\times 2$), allowing for some flexibility and a less constrained analysis. This ensures that every variable contributes, even if its contribution is the minimum, ${w}_{j}$. While alternative values were explored, selecting a value lower than this would lead to an overly unrestricted analysis, which is impractical in the context of healthcare management units. Conversely, opting for a larger value would reduce the flexibility of the optimization model in assigning weights.

### Characterization of the software used

Regarding the coding, implementation, computation of the model, and the missing data treatment, all were done through MATLAB R2022b. Upon appropriate justification, the codes used for implementing the Benefit-of-the-Doubt approach and the imputation/simulation techniques can be made available to interested parties.

## Findings and discussion

This section presents and discusses the experimental results obtained, structured according to the research questions stated in the introduction. The results demonstrate the evolution of performance of the entities over time, the identification of benchmark hospitals and their changes in ranking, the identification of the best-prepared hospitals for extreme cases, and an evaluation of the advantages and disadvantages of the employed models.

### The impact of the pandemic on the Portuguese public hospitals’ performance

For each model, 5000 CI scores were generated for every entity due to missing data imputation. The expected value of these scores was then computed and employed in subsequent analysis. It is worth noting that, for entities with no missing data, the values remain fixed and consistent; thus, the average score represents the observed value.

**Fig. 1:**
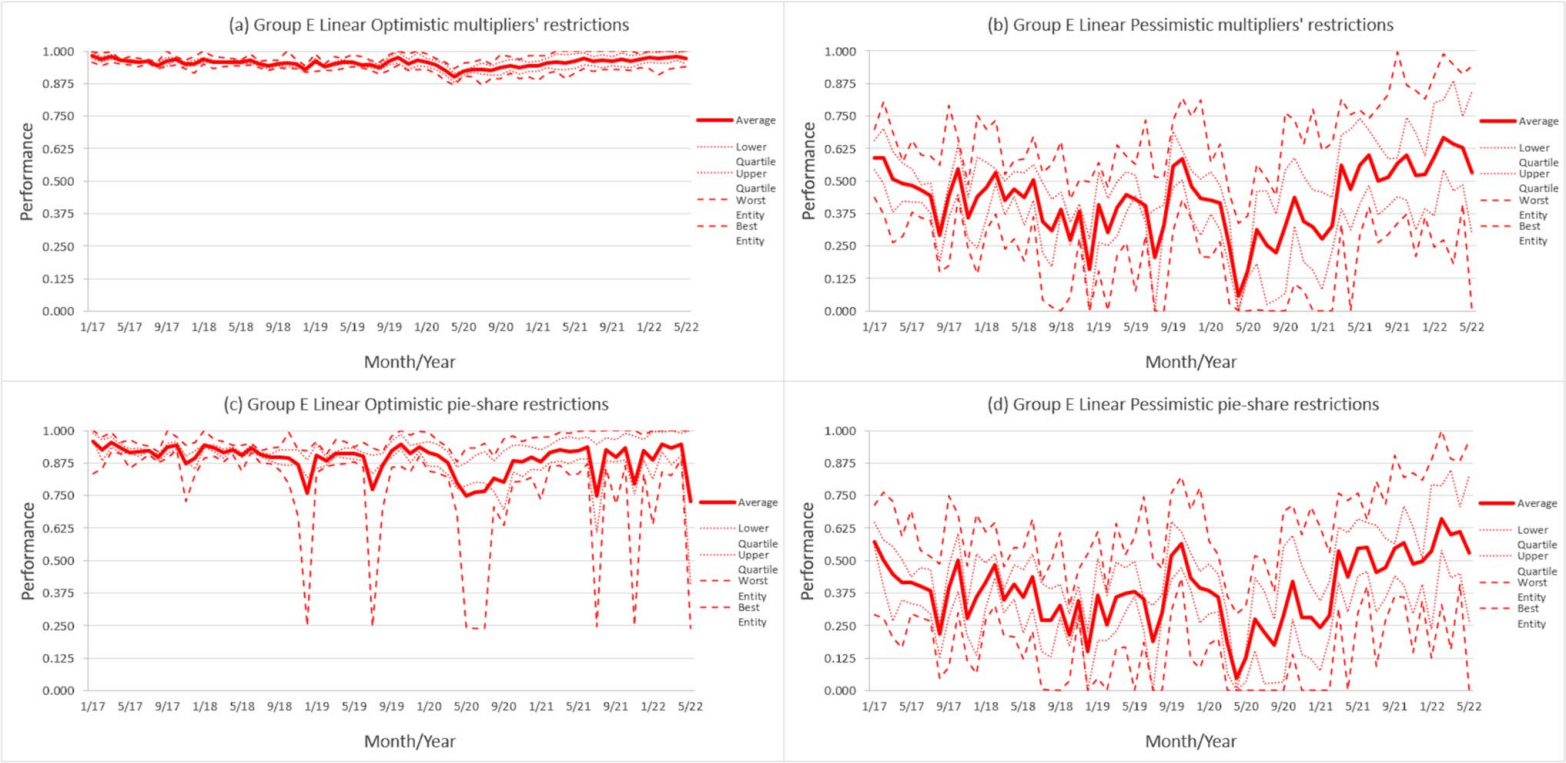
Evolution of performance scores for each model for group E

Figure 1 illustrates the evolution of the average CI score for each model within group E (Group E was chosen for its inclusion of the largest and most complex hospitals with substantial funding), based on the ACSS clustering, spanning from January 2017 to May 2022 (groups B, C, and D are depicted in Figures A1, A2, and A3 in the Appendix, respectively). Each figure includes four graphs, with one corresponding to each model (optimistic/pessimistic) and restriction (multiplier/variable). The graphs have been designed to showcase five lines, resulting in a clear and concise display of the distribution, tendency, and variability of the performance scores. The central value for each month, which is the average of each group throughout the period, is indicated by a bold and thick line. The dashed lines, which represent the minimum and maximum values, show the range of scores for each group, highlighting the best and worst scores for each month. Lastly, the first and third quartiles, displayed in dotted thin lines, offer information about the data range, and how isolated the minimum and maximum scores are from the rest of the scores. Through these lines, it is possible to gain insight of whether changes in the group's average are primarily due to improvements/declines among top/bottom performers, or if they are the result of a more uniform change across the entire group.

The stated graphs suggest certain assumptions that will be thoroughly tested, verified, and explained to ensure their reliability. The objective of this process is to validate whether these assumptions are accurate or not.

Firstly, regardless of the specific model and group being considered, there is always a significant decline in performance scores during April 2020, which overlapped with the peak of the first wave of the COVID-19 pandemic when there was widespread fear and uncertainty. This drop can be attributed to a major degradation in the values related with access variables, characterized by longer delays, excessive inpatients, and lack of staff, among others, caused by the implementation of full and strict lockdown measures. Subsequent waves of the pandemic have been associated with smaller decreases in average performance scores, due to the fact that governments, hospitals, and the general public have since established guidelines and were better prepared to manage the impacts of the pandemic leading to less stringent lockdowns measures and therefore the access variables were not as severely affected. As a result, there have been fewer disruptive changes that affected the performance of the entities during subsequent outbreaks.

Accurately quantifying the exact impact of the pandemic on performance scores is a challenging task. However, both optimistic and pessimistic approaches have highlighted noticeable drops in average scores during the pandemic period. Given that these approaches represent respectively the maximum and minimum values of each groups’ performance, indicates that there was indeed a reduction in these scores. Thus, it is reasonable to conclude that the pandemic had a significant impact on performance scores.

From the onset of the pandemic, a discernible and consistent trend towards improvement has been observed, as evidenced by the findings initially implied by Nunes & Ferreira [30]. This trend, characterized by entities achieving scores surpassing pre-pandemic levels, raises intriguing questions about the underlying factors driving such enhancements. One plausible explanation for this phenomenon lies in the observed reduction in new patients seeking medical care during the pandemic [79–81]. Consequently, the apparent increase in performance scores could be attributed, at least in part, to a decrease in patient volume rather than an actual enhancement of hospital quality [82, 83]. With fewer patients, hospitals experience shorter wait times and increased appointment availability, positively impacting access-related metrics. Additionally, lower patient loads alleviate strain on resources, enhancing operational efficiency. This phenomenon is not consistent across all entities, as will be examined in detail in Sect. 5.3, where the underlying causes will be discussed.

### Exploring the consistency of benchmark entities pre and post COVID-19 outbreak

In DEA, benchmarks are considered the efficient entities because they represent the best possible performance that can be achieved by a DMU given a specific set of inputs and outputs. The benchmark DMUs are used as reference points to evaluate the efficiency of other DMUs. By comparing their efficiency scores to the benchmark, inefficient DMUs can identify areas where they can improve their performance and become more efficient. It is worth noting that in DEA, there can be multiple benchmarks depending on the specific inputs and outputs used to evaluate the efficiency of the DMUs. Therefore, the choice of benchmark can have a significant impact on the results of the analysis.

In this case study, there was not a constant benchmarking entity as the benchmark varied over time for each group. Therefore, instead of selecting only one benchmark entity for each group, an analysis was conducted to identify the top entities (which refer to the entities with the highest performance scores) in each group and how often they were considered as benchmarks before the COVID-19 outbreak. This analysis aimed to understand their stability as benchmarks following the outbreak. Table 2 provides information on this sensitivity analysis, detailing how frequently benchmark entities (the top performers) ranked among the top one to five entities across the pre-pandemic months.

**Table 2:** Proportion of months in which benchmarks rank among the top one to five entities before the COVID-19 outbreak

No. of Benchmarks	Group B	Group C	Group D	Group E
1	59%	36%	49%	36%
2	92%	56%	67%	51%
3	100%	72%	74%	77%
4	100%	80%	90%	90%
5	100%	90%	97%	95%

This shows, for example, that the top two entities in Group E are considered benchmarks in 20 out of the 39 months analysed, as the percentage is 51% (from January 2017 to March 2020, inclusive). This indicates that in roughly half of the months examined, the benchmark is one of the top two entities selected.

Additionally, Table 3 was developed to assess the impact of the pandemic on the performance and consistency of top-performing entities. Specifically, it examines whether the pandemic led to an increase, decrease, or no change in the performance score and frequency with which each entity served as benchmarks for performance.

**Table 3:** The differences in performance for each entity, before and after the start of the pandemic, in group E. The two best entities are in bold

Group E entities	Average performance pre-March 2020	Average performance post-March 2020	Time considered benchmark pre-March 2020 (%)	Time considered benchmark post-March 2020 (%)
Centro Hospitalar Universitário Lisboa Norte, EPE	0.962	0.962	26%	15%
Centro Hospitalar Universitário de Lisboa Central, EPE	0.953	0.957	13%	4%
**Centro Hospitalar Universitário de São João, EPE**	**0.966**	**0.976**	**36%**	**42%**
**Centro Hospitalar Universitário do Porto, EPE**	**0.963**	**0.980**	**15%**	**46%**
Centro Hospitalar de Lisboa Ocidental, EPE	0.940	0.914	5%	0%
Centro Hospitalar e Universitário de Coimbra, EPE	0.948	0.924	5%	0%

The choice of group E for this illustration was intentional because of its smaller size, resulting in a more concise and easily interpretable table. Group E consists of only six entities, with two recognized as top performers and 4 as the underperformers. It is important to note that this analysis was carried out across all groups to provide comprehensive insights, as shown in Table 8. Additionally, group E holds significance as it comprises some of the largest and most complex hospitals, which receive substantial funding.

As shown in Table 3, the two top-performing entities in group E remained the leading entities after the pandemic. This trend is observed across all groups, as seen in Table 8. Furthermore, before the pandemic, the top two entities in each group had an average combined benchmark frequency of 66.5%, with group E being the minimum at 51%. After the pandemic, the top two entities remained the most frequently considered benchmarks, but now with an average frequency of 83.5%, with the lowest at 65% in group B, a significant increase of over 25%.

### Differential impacts of the pandemic on top performers and underperforming entities

Although the later stages of the pandemic have shown an improvement in the average performance of the groups, surpassing pre-pandemic levels, a closer analysis of Figs. 1, 4, 5, and 6 suggests that this improvement was not uniform across all entities. The top-performing entities demonstrated a more rapid and substantial increase in performance after the pandemic when compared to the lower-performing ones, as indicated by the dashed and dotted lines in the aforementioned figures. This observation is further supported by Table 3 and Table 8, which reveal that the top entities not only saw improvements in their performance scores but were also considered benchmarks more frequently. In contrast, the worst-performing entities generally experienced a decline in their scores and a reduction in their respective frequency of being considered a benchmark, indicating that they were significantly more affected by the pandemic.

As seen in Table 3, the two top-performing entities in group E were the only ones to experience an improvement in performance following the pandemic. Furthermore, both entities saw an increase in the frequency with which they were regarded as benchmarks. In contrast, the remaining entities, which had lower initial performance scores, were generally negatively impacted by the pandemic, being selected as benchmarks less frequently and often with reduced performance scores.

Notably, across all groups, as shown in Table 8, a clear pattern emerges: the top two entities improved their performance following the COVID-19 outbreak. These entities also remained the most frequently identified benchmarks, surpassing the 50% threshold in the post-pandemic period. With the exception of Group B, the top entities in each group saw an increase in the frequency of being considered a benchmark after the pandemic. Group B, while an exception, where the frequency of the top two entities being recognized as benchmarks decreased, they still both held the highest frequency, exceeding the 50% mark with a value of 65%. This highlights the consistency and resilience of these top performers.

**Fig. 2:**
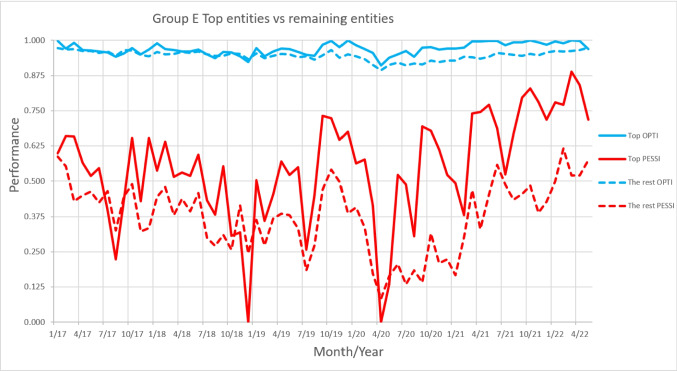
Performance comparison of top entities versus underperforming entities under optimistic and pessimistic approaches for Group E

**Fig. 3:**
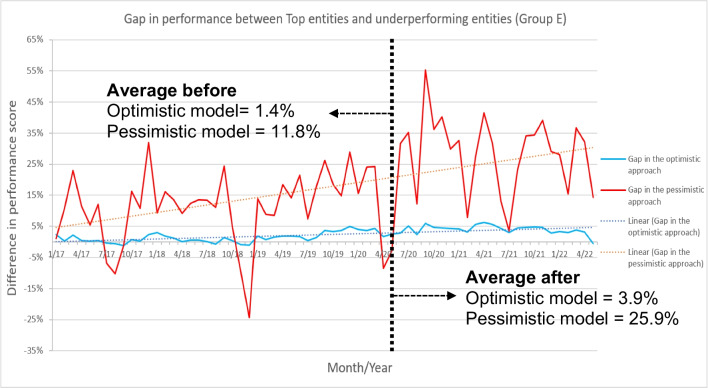
Performance gap between benchmarks and lowest-performing entities, with the average gap before and after the pandemic, within Group E. Trendline with positive slope in both approaches represented with a dotted line

**Fig. 4:**
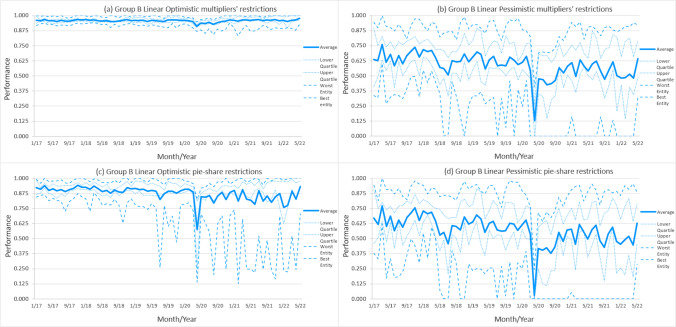
Evolution of performance scores for each model for group B

**Fig. 5:**
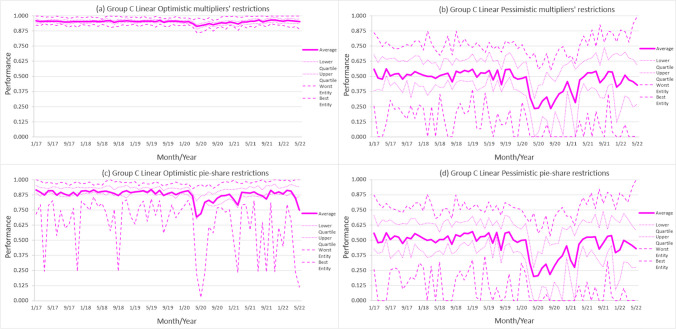
Evolution of performance scores for each model for group C

**Fig. 6:**
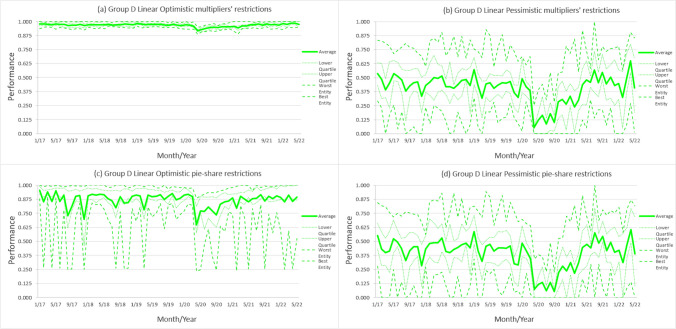
Evolution of performance scores for each model for group D

This shows a significant discrepancy in how entities were affected by the pandemic, seemingly associated with their respective performance levels. To better understand this discrepancy, Fig. 2 illustrates the contrasting behaviours of the top entities versus the underperforming entities of Group E. Additionally, Fig. 3 portrays the monthly performance difference between the benchmarks and the underperforming entities, considering both optimistic and pessimistic approaches also within Group E.

Figure 2 demonstrate that the performance gap between the top and underperforming entities increased after the pandemic outbreak, meaning that the top entities mainly drove the increase in performance, rather than an overall improvement across all entities. This trend is evident in both the optimistic approach (indicated by the blue lines) and supported by the pessimistic approach (represented by the red lines). These results are also highlighted on Fig. 3, where the gap is quantified. On average, both perspectives observed a widening gap following the start of the pandemic, marked by the vertical dashed black line. Additionally, the trendlines in both cases display a positive slope, signifying that the gap has continued to widen over time.

This gap between the top-performing entities and the rest of the entities remained consistent across all groups, and notably larger after the pandemic outbreak. For these top entities, the recovery from COVID-19 was not only faster and sharper but brought much more resilience into the next waves. The scores of the top entities were better, more tenacious, and with less deviations between them, as seen in Fig. 2. This finding suggests that the top entities may have developed greater adaptability and resilience to external shocks over time. These differences must be identified to provide insights into how entities can build resilience and withstand future shocks.

Nevertheless, in some instances, driven by new waves of COVID-19, there were significant declines in performance scores, which temporarily changed the distance separating the best from the worst entities. In particular, there are cases where the underperformers outperformed the top entities. A deeper examination of the raw data shows that this shift in rankings is closely linked to the clinical safety set of variables. In most months, nearly all entities achieve the optimal or close to the optimal outcome—reporting zero adverse events per 1,000 cases (or whatever the variable's standard might be). As a result, when an adverse event does occur, the corresponding values sharply increase, due to the standardization process. While this impact isn’t drastic, given the presence of other variables, it is significant enough to cause lower-performing entities to occasionally surpass the top performers. These findings highlight that even the best-performing entities can face challenges, and under certain circumstances, lower-performing entities may outperform them.

The main takeaway from this subsection is that, in every group, the top two entities prior to the pandemic consistently experienced an improvement in their performance scores following the outbreak. Additionally, their frequency of being recognized as benchmarks increased. In contrast, the lower-performing entities, which were rarely considered benchmarks, generally faced a significant decline in both their performance scores and the frequency of being identified as benchmarks. The exception is Group B, where the top entities, while still showing improved performance, did not see a rise in their benchmark frequency, though they still were the most frequent ones.

### Policy implications

The existing methodology employed by the Portuguese government for hospital performance analysis offers a limited perspective, lacking a comprehensive view or a ranking system for individual hospitals. This study introduces methods that enable the identification of top-performing entities. By identifying resilient and benchmark institutions that maintained or improved their performance despite pandemic challenges, underperforming hospitals can potentially enhance their capabilities for future crises by recognizing the disparities between them. This section will discuss the key implications across the four dimensions analyzed.

#### Access

During the pandemic, hospitals faced stringent access restrictions, resulting in a substantial drop in patient treatment volumes and overall performance. Fear of infection further limited patient access to healthcare services, highlighting a critical area for improvement in future crisis management. Policies should focus on maintaining public confidence in healthcare systems during health emergencies.

An analysis of the leading hospitals' annual reports from 2020 onward reveals a shared strategy of anticipation and preparation. These top-performing entities were proactive in their decision-making, taking preemptive measures to prepare for the pandemic. As early as January 2020, two months before the first confirmed COVID-19 case in Portugal, these top entities began implementing strategies such as reorganizing spaces and expanding reserves of medical supplies and equipment. This preparedness allowed them to weather the crisis more effectively. Policymakers should encourage and support similar proactive strategies across the healthcare system.

#### Productivity

Although hospital expenses remained constant during the pandemic, overall productivity declined due to the sharp drop in patient output and the strain on resources. However, this impact was not uniform across all hospitals. The top-performing entities, which had already established strategic advantages before the pandemic, experienced smaller productivity declines and were able to recover more quickly. Their superior performance can be attributed to pre-existing operational advantages, including efficient resource management and strategic foresight.

The findings suggest that simply increasing funding during a crisis does not necessarily lead to improved productivity or efficiency. In fact, as noted by Olsen et al. [6], increased funding without strategic resource utilization can exacerbate inefficiencies. Therefore, long-term policies should focus on optimizing resource use, rather than simply increasing budgets.

#### Clinical safety and care appropriateness

The research highlights that while all entities were able to uphold high clinical safety standards, underperforming hospitals experienced more fluctuations in safety metrics such as adverse event rates.

It is essential to consider healthcare quality alongside productivity when assessing hospital efficiency. The number of cases treated alone does not fully capture a hospital’s performance. Instead, healthcare efficiency should be evaluated based on patient outcomes, including recovery and well-being, rather than focusing solely on throughput. According to Ferreira et al. [36], achieving excellence in healthcare hinges on meeting these standards, ensuring that hospitals are both efficient and effective without creating barriers to access. The pandemic underscored the need for a more comprehensive approach to measuring healthcare performance, one that balances both efficiency and quality of care.

## Conclusions

This paper studies the impact of COVID-19 on the efficiency and performance of the Portuguese public hospitals, investigating whether that impact was generalized across all entities or if there were any discernible characteristics or policies which influenced this impact. To evaluate this, the Benefit-of-the-Doubt approach was used, to address the major problem of multiple criteria decision analysis methods, which has the challenge of reaching a consensus when assigning the set of weights for each variable. These models overcome data imperfections as well as account for both desirable and undesirable outputs, proving to be a robust and effective tool for real world conditions.

Firstly, the results show that every COVID-19 wave led to a decline in the performance, with the first wave being drastically more impactful than subsequent ones, since there was no knowledge about the pandemic and no preventive measures in practice. Notably, this initial drop affected even the variables where hospitals had previously excelled. Following the first sharp decline, there was a clear tendency of improvement, with some entities achieving higher scores than pre-pandemic heights, firstly implied by Nunes & Ferreira [30]. This improvement may be attributed to the reduction of new patients, possibly driven by patients' concerns about the virus, rather than an actual improvement of the hospital’s qualities, as stated by the cited authors.

Secondly, the top two entities from the pre-pandemic period in each group remained unchanged throughout the pandemic, continuing to be the most frequently identified benchmarks within their respective groups. Before the pandemic, their average benchmark frequency was 66.5%, which increased to 83.5% post-pandemic, representing a rise of over 25%. This indicates that not only did the top entities maintain their status as the most frequent benchmarks during the pandemic, but they also strengthened their dominance. They outperformed before the pandemic and continued to do so despite the challenges posed by it, underscoring their resilience and consistency.

Thirdly, the top two entities in each group always improved their performance after the pandemic outbreak, in relation to their pre-pandemic levels. In contrast, lower-performing entities generally experienced a decline in both performance scores and benchmark frequency. The performance gap between the top and underperforming entities widened after the pandemic, driven primarily by the stronger recovery of the top entities, which demonstrated greater resilience and adaptability to external shocks.

However, this methodology has inherent limitations. It is highly sensitive to both the sample size and the selected variables. Alterations in either can lead to different results, as adding or removing benchmark entities may significantly shift the efficient frontier, altering the performance values. Furthermore, the BoD linear approach has a compensatory nature since subpar scores in some variables may be offset by strong performances in others.

In the near future, the compensatory nature of the models is expected to be addressed through a geometric aggregation approach, as demonstrated by Ferreira et al. [17] and initially suggested by Cherchye et al. [20]. Additionally, the Hit-and-Run method will be employed for simulating missing data, which may yield more accurate and realistic results [84]. Efforts will also focus on gathering input from policymakers on three key aspects: determining minimum weights for each variable, developing value functions instead of using simple linear rescaling, and their preferred methods for handling missing data.

## Data Availability

The data used in this study are sourced from official records accessible at: https://benchmarking-acss.minsaude.pt/BH_AcessoDashboard.

## Appendix

See Table 4, Table 5, Table 6, Table 7 and Table 8.

**Table 4:** Literature Review on healthcare management units and the impact of the COVID-19 pandemic

**Author(s) and study**	**Brief Description of the study**
Gourinchas, [25]. Flattening pandemic and recession curves	Gourinchas starts by pointing out that the capacity of any country’s health system is limited. If that upper limit is exceeded, the pandemic would disrupt any health system, leading to a severely bigger mortality risk. To prevent that, countries had to impose policies to “flatten the pandemic curve”. The author argues that these policies, lead to substantial economic damage with excessive deterioration in GDP numbers. Gourinchas defends that urgent macroeconomic support is needed but the measures that help the economy, are counterproductive towards the health crisis, and vice versa
Breitenbach et al., [7]. Global healthcare resource efficiency in the management of COVID-19 death and infection prevalence rates	In their study, Breitenbach et al. [7] dive into the efficiency analysis of healthcare during COVID-19. The authors employed a DEA approach which takes into consideration desirable and undesirable outputs under the variable returns to scale hypothesis. The results showed a very low overall efficiency level handling the pandemic and even a reduction of the efficiency during this period. The author argues that this is because countries tried to combat the pandemic by “throwing” resources at it, lowering the efficiency by wasting inputs. The efficient countries showed that utilizing resources effectively is more valuable than simply having more resources
Mirmozaffari et al. [29]. A novel hybrid parametric and non-parametric optimisation model for average technical efficiency assessment in public hospitals during and post-COVID-19 pandemic	This paper proposes a new model by averaging the results from DEA, a non-parametric method, and SFA, a parametric method, to evaluate hospital efficiency during the first six months of the COVID-19 pandemic. The justification for this apparently contradictory approach is to counteract each method’s weaknesses and strengths by integrating both approaches. This method was used to determine the most efficient and inefficient hospitals and areas of improvement were identified. The paper highlights the deficiencies in patient care during the pandemic and suggests future research combining other parametric and non-parametric methods, and running verified simulation models for potential robustness testing
Seo et al. [33]. Impact of the COVID-19 pandemic on mental health service use among psychiatric outpatients in a tertiary hospital	This study analysed the impact of the COVID-19 pandemic on outpatient clinic use in a tertiary hospital for different psychiatric diagnoses. The results showed significant reductions in mental health service use during the pandemic. The study suggests that patients with anxiety or depression may have more concerns about the spread of COVID-19 and be less likely to visit outpatient clinics. The study recommends considering alternative service delivery methods such as telepsychiatry during a pandemic
Ornell et al. [32]. The next pandemic: impact of COVID-19 in mental healthcare assistance in a nationwide epidemiological study	The authors found that the COVID-19 pandemic has resulted in a significant decline in mental health consultations and group interventions. This decline has resulted in an increase in emergency assistance, which may lead to a parallel pandemic of mental health issues that may last for a long time. Patients who were already receiving mental health care may have had their treatment interrupted, and this can lead to an increase in the global burden of disease. The study also found that there might be a relationship between an increase in some treatment modalities and a decrease in others during the COVID-19 pandemic. The study recommends the adaptation of existing mental services to mitigate the negative effect of the pandemic on mental health breakdown
Lupu & Tiganasu [28]. COVID-19 and the efficiency of health systems in Europe	Lupu & Tiganasu [28] evaluated the efficiency of health systems of 31 European countries in three different phases: the first wave, the relaxation period and the second wave. On a later stage, Tobit type regression was used to measure the impact of each dimension on the efficiency. The findings showed that there are several aspects which influence the efficiency of health systems; for example, the psychological effect from the fear of catching the disease led to less appointments, which led to an overall bigger number of unchecked diseases. The authors conclude that for a good response against the epidemic, it is crucial a cooperation between the population, by complying with the restrictions, and the government/authorities, by developing strategies
Shah et al. [27]. A qualitative analysis of psychosocial stressors and health impacts of the COVID-19 pandemic on frontline healthcare personnel in the United States	Shah et al. provided a qualitative study that explored the experiences of frontline healthcare professionals (HCP) in the early stages of the COVID-19 pandemic in the United States. The study found that HCP experienced a range of work-related psychosocial stressors and highlights the need for workplace and community support for frontline HCP, particularly during times of crisis
Duden et al. [31]. Global impact of the COVID-19 pandemic on mental health services: A systematic review	The authors examined how mental health services have been affected by the COVID-19 pandemic. The results showed that the pandemic has had significant impacts on mental health services, with disruptions, shortages of staff and equipment, and transitions to virtual or hybrid care models. Studies have shown increases in mental health problems and caseloads, and efforts are needed to support staff and prevent burnout as mental health services are essential for vulnerable populations during a crisis and should receive specific attention in pandemic regulations
Nunes & Ferreira [30]. Evaluating Portuguese Public Hospitals Performance: Any Difference before and during COVID-19?	In their study, Nunes and Ferreira examined the efficiency and effectiveness of hospital healthcare providers in Portugal, both before and during the COVID-19 pandemic. They discovered that the efficiency dimension experienced a decline in 2020 due to a decrease in hospital production, but it resumed an upward trend in 2021. Despite the exodus of highly qualified health professionals in the pre-pandemic period, the effectiveness dimension showed a continuous growth trend during the pandemic period in all groups. Although the pandemic resulted in an increase in barriers to access, vaccination efforts helped reduce the severity of the disease. While some quality variables exhibited poor results, hospitals made progress in other areas, which compensated for the shortcomings. These findings provide hope and assurance regarding the sustainability of the Portuguese national health system
Henriques & Gouveia [9]. Assessing the impact of COVID-19 on the efficiency of Portuguese state-owned enterprise hospitals	Henriques & Gouveia examined the efficiency and productivity of Portuguese state-owned enterprises (SOE) hospitals before and after the COVID-19 pandemic using data from the NHS. The study uses the VBDEA framework to handle DMs' preferences and considers two types of value functions reflecting different perspectives towards the pandemic context. The results show that 21 and 17 out of 37 SOE hospitals were efficient in 2019 and 2020, respectively. The study finds that the average hospital efficiency decreased by 61% from 2019 to 2020 under a neutral perspective, and by 100% under a conservative perspective adopted by a real DM. The negative impact of COVID-19 on hospital activity levels was more pronounced in efficient than in inefficient hospitals, implying that COVID-19 had less of an impact on inefficient units' activity levels but at the price of a significant increase in their level of total resources. The study suggests that hospitals should operate adjustments that are beneficial regardless of the hurdles they face and foster a culture of cooperation within and across hospitals to become more resilient even for future COVID-19 outbreaks

**Table 5:** Summary of Recent Applications of Data Envelopment Analysis (DEA) in the Portuguese Healthcare System

**Author(s) and study**	**Brief Description of the study**
Simões & Marques [85]. Performance and congestion analysis of the Portuguese hospitals’ services	Simões and Marques conducted a strict efficiency analysis to evaluate the performance of 68 hospitals in Portugal in 2005. The study utilized the DEA approach and incorporated a double-bootstrap procedure to account for the influence of the operational environment on efficiency. The results of the study provide valuable insights into the relative efficiency of the hospitals, where significant levels of inefficiency were found, and highlight potential areas for improvement. Overall, the study highlights the importance of utilizing advanced analytical tools and techniques to optimize performance and enhance the quality of healthcare services
Ferreira et al. [13]. On evaluating health centers groups in Lisbon and Tagus Valley: efficiency, equity and quality	Ferreira et al. focused on evaluating the performance of Primary Health Care (PHC) providers in the Lisbon and Tagus Valley region of Portugal, considering efficiency, quality, and equity. To measure efficiency, the non-parametric data envelopment analysis (DEA) method was employed, along with the partial frontier method of order-m to assess the impact of exogenous variables. Analysis underscores the importance of PHC efficiency, given its cost-effectiveness potential. Exogenous variables like distance to the nearest hospital positively influence efficiency, while factors like purchasing power, aging population, and population size negatively affect it. The study concludes that optimizing resource usage, fostering a learning network, and disseminating best practices can enhance efficiency without compromising quality and equity
Marques & Carvalho [86]. Estimating the efficiency of Portuguese hospitals using an appropriate production technology	Marques and Carvalho examined the production technology employed by hospitals in Portugal using statistical tests. The paper emphasizes the significance of technology assessment prior to estimating efficiency levels. The research was conducted over a four-year period (2005–2008) and involved the application of two different models: one based on monetary units and the other on quantities. The study concludes that the production technologies utilized by Portuguese hospitals exhibit globally non-increasing returns to scale with a 95% confidence interval
Castro et al. [46]. Benchmarking dos serviços dos hospitais portugueses: uma aplicação de data envelopment analysis	Castro et al. presented DEA applied to most hospitals of the Portuguese NHS, with a set of inputs and outputs. The model has weights restrictions which lead to more feasible coefficients and therefore more feasible proposals to better the efficiency of their entities. Finally, the authors indicated exactly where possible improvements should be made to have a bigger impact on the efficiency of the entities. The set of input and output variables is very well argued, and initial *weak* weight restrictions were set to allow the DMUs room to work
Almeida [87]. Evaluating Hospital efficiency adjusting for quality indicators: an application to Portuguese NHS Hospitals	Almeida sought a methodology to incorporate quality indicators in the analysis of efficiency. The quality was measured through patient surveys and efficiency with the DEA approach. The authors discovered that entities which were efficient, remained efficient, almost regardless of the weight assigned to the quality indicators; entities which were inefficient, suffer an impact when adjusted for quality. According to the authors, there may be a trade-off between efficiency and quality however, it is possible to increase efficiency without lowering the quality level
Ferreira & Marques [49]. Did the corporatization of Portuguese hospitals significantly change their productivity?	Ferreira & Marques explored if the reforms in the Portuguese health system had an impact in performance and productivity of the NHS hospitals. The entities were analysed for the period between 2002 and 2009. The authors concluded that the hospitals which did not experience any reform, were the ones with higher performance, and that performance was inversely correlated to efficiency
Ferreira & Nunes [88]. Technical efficiency of Portuguese public hospitals: A comparative analysis across the five regions of Portugal	This paper aims to compare the efficiency between twenty-seven hospitals across five different Portuguese health administrative regions using DEA. The results showed that, on average, the performance is high among all areas, but with clear regions outperforming. The authors pointed out that Alentejo and Lisbon regions were the worst performers, presenting possible improvements
Kohl et al. [41]. The use of Data Envelopment Analysis (DEA) in healthcare with a focus on hospitals	Kohl et al. reviewed 262 papers of DEA applied to healthcare, including the ones mentioned, to identify the broader trends and offer specific insights. The authors covered a period of over ten years and analyzed the research goals, methodological settings, and models applied in the studies. The authors group the research goals into four clusters and provide descriptive statistics of the papers. Additionally, the paper serves as a roadmap to important methodological literature and publications, offering valuable insights for researchers planning to apply DEA in a hospital setting and potential strategies on how to reach managers and policymakers
Matos et al. [48]. Economic analysis of Portuguese public hospitals through the construction of quality, efficiency, access, and financial related composite indicators	Matos et al. [48] analysed the efficiency of Portuguese public hospitals between 2013 and 2017. The dimensions that went into consideration are Quality, Efficiency, Access, and Financial to understand what contributes to a higher performance. The authors conclude that the financial aspect has a higher overall score than the quality aspect, putting in question whether these hospitals are run with focus on quality and access or with focus on financial results

**Table 6:** Literature Review on the linear BoD approach

**Author(s) and study**	**Brief Description of the study**
Melyn & Moesen [58]. Towards a synthetic indicator of macroeconomic performance: Unequal weighting when limited information is available	The study proposes a methodology for constructing a synthetic indicator of macroeconomic performance when limited information is available. The methodology uses unequal weighting based on the relative importance of each variable in the overall macroeconomic performance. The authors apply the methodology to Belgium and compare it to other existing methods. They find that the synthetic indicator constructed using their proposed methodology performs better in terms of predictive power and robustness. They also discuss the implications of unequal weighting in constructing synthetic indicators and highlight the importance of choosing the appropriate weights for each variable
Cherchye et al. [20]. An introduction to ‘benefit of the doubt’ composite indicators	Cherchye et al. [20] propose the BoD approach to aggregate key performance indicators (KPIs) into a score with no information about the inputs. Each entity will have a single score called CI which is equal to the maximum weighted arithmetic mean, with entity specific weights. These weights are subjected to constraints which forces all the other entities CIs to be equal or lower to one with each entity specific set of weights. This approach allocates higher weights to KPIs where the entity performs well and lower weights to KPIs where the entity performs worse, thus arriving to the best possible score. Since the authors do not know the weights with each entity evaluates itself, they are deduced by looking at the strengths and weaknesses of each, making the CI value as high as possible, giving each entity the Benefit-of-the-Doubt. Since the CI value is based on an arithmetic mean, the approach is compensatory and because it gives the best value possible, it is also very generous
Zhou et al. [51]. A mathematical programming approach to constructing composite indicators	Similarly to Cherchye et al. [20], the authors aggregate the KPIs into a score of performance for each entity. However, instead of looking for the best possible score, a conservative approach was taken, choosing the worst-case scenario. In the end, Zhou et al. [51] join their CI score with Cherchye et al. [20] score through a linear scaling between the maximum and the minimum values. This linear scaling is done through a parameter which weights how much each score contributes to the final value depending on how benevolent or unkind the DMU wants to be
Guardiola & Picazo-Tadeo [52]. Building weighted-domain composite indices of life satisfaction with data envelopment analysis	Guardiola et al. (2014) suggest a combination between the BoD and multicriteria decision analysis resulting in an equal set of weights for all entities. The authors believe that comparing entities with different sets of weights is not fair, which is one of characteristics of the BoD approach. Furthermore, the authors defend that it is more sensible that DMUs have the right to choose the weight of each dimension regarding themselves. The BoD approach is able to incorporate this by adding weight restrictions which are more flexible and allow for a better interpretation
Zanella et al. [54]. Undesirable outputs and weighting schemes in composite indicators based on data envelopment analysis	Zanella et al. [54] tackle the problem of having desirable and undesirable outputs, which are variables that need to be maximized while others need to be minimized. The authors consider two approaches: direct and indirect. The first, is based on a directional distance function model, allowing for the original data to be used without prior treatment. The second, requires a transformation on the scale of the raw data to turn the undesirable variables into maximization outputs
Fusco et al. [55]. Spatial heterogeneity in composite indicator: A methodological proposal	Fusco et al. highlight the importance of properly considering the spatial heterogeneity of units when computing CIs to describe complex systems. The authors introduce an additional constraint associated with spatial proximity to the robust BoD framework. The paper provides two empirical illustrations to highlight the heterogeneous contributions of external factors in explaining performance differential of a unit among different points in space. The paper suggests future research should focus on identifying spatially homogeneous optimal cluster of points
Färe et al. [56]. A benefit-of-the-doubt model with reverse indicators	Färe et al. [56] present another solution to implement the undesirable KPIs, which are indicators that are not constant, and their increasing values are considered undesirable events, in the model and criticize the previous presented solutions. The authors show that the dual formulation of the BoD model can incorporate undesirable KPIs by treating them as inputs. This enables an increase in selected KPIs while decreasing the remaining ones
Ferreira & Marques [47]. Public–private partnerships in health care services: Do they outperform public hospitals regarding quality and access? Evidence from Portugal	Ferreira & Marques [47] proposed a non-radial directional BoD model that returns a set of slacks, either positive or negative for desirable or undesirable variables, respectively. This approach is more flexible than the commonly used radial model and allows for an easier interpretation of the inefficiency due to the slacks. The slacks in a non-radial model represent the amount by which a DMU can improve its performance on a particular input or output without worsening its performance on any other input or output
Matos et al. [48]. Economic analysis of Portuguese public hospitals through the construction of quality, efficiency, access, and financial related composite indicators	Matos et al. [48] studied the reasons behind low performance and high indebtedness levels in Portuguese public hospitals. For that, the authors used the Benefit-of-the-Doubt approach to obtain CI score for each of the following dimensions: Quality, Efficiency, Access, and Financial. After that, the Benefit-of-the-Doubt approach was used again on the CI scores of each dimension, obtaining a final CI score from a weighted arithmetic mean of every dimension. This model ends up with scores that may be too benevolent due to the application of the BoD method twice
Fusco [53]. Potential improvements approach in composite indicators construction: The Multi-directional Benefit of the Doubt model	Fusco proposes a new method called "Multi-directional BoD" (MDBoD) for constructing Confidence Intervals (CIs) based on a frontier approach. This method allows for the introduction of non-compensability among simple indicators in CI construction in an objective manner. From an economic point of view, MDBoD enables the distinction of units with weak or strong CI scores and provides practical advice on how units can improve their performance. Possible enhancements of the method include the introduction of contextual variables through conditional or spatial measures to differentiate fully efficient units
Ferreira et al. [17]. A geometric aggregation of performance indicators considering regulatory constraints: An application to the urban solid waste management	The paper proposes a new model, the Multiplicative BoD model, for constructing CIs that account for regulatory constraints, undesirable KPIs, and imperfect data knowledge. The model introduces regulation-required constraints that impact entities' performance assessments, which have been disregarded in previous research. The framework constructs a frontier such that all targets with respect to it obey all constraints, and it can be straightforwardly adapted to any performance analysis featuring imperfect data knowledge

See Fig. 4, Fig. 5 and Fig. 6

**Table 7:** List of considered entities and the corresponding group

Group	Entity
Group B	Centro Hospitalar Póvoa de Varzim/Vila do Conde, EPE
	Centro Hospitalar do Médio Ave, EPE
	Centro Hospitalar do Oeste, EPE
	Hospital Distrital da Figueira da Foz, EPE
	Hospital Santa Maria Maior, EPE
	Unidade Local de Saúde da Guarda, EPE
	Unidade Local de Saúde de Castelo Branco, EPE
	Unidade Local de Saúde do Litoral Alentejano, EPE
	Unidade Local de Saúde do Nordeste, EPE
Group C	Centro Hospitalar Barreiro/Montijo, EPE
	Centro Hospitalar Entre Douro e Vouga, EPE
	Centro Hospitalar Médio Tejo, EPE
	Centro Hospitalar Tâmega e Sousa, EPE
	Centro Hospitalar Universitário Cova da Beira, EPE
	Centro Hospitalar de Leiria, EPE
	Centro Hospitalar de Setúbal, EPE
	Centro Hospitalar do Baixo Vouga, EPE
	Hospital Distrital de Santarém, EPE
	Hospital da Senhora da Oliveira, Guimarães, EPE
	Hospital de Cascais, PPP
	Hospital de Loures, EPE
	Hospital de Vila Franca de Xira, EPE
	Unidade Local de Saúde de Matosinhos, EPE
	Unidade Local de Saúde do Alto Minho, EPE
	Unidade Local de Saúde do Baixo Alentejo, EPE
	Unidade Local de Saúde do Norte Alentejano, EPE
Group D	Centro Hospitalar Tondela-Viseu, EPE
	Centro Hospitalar Trás-os-Montes e Alto Douro, EPE
	Centro Hospitalar Universitário do Algarve, EPE
	Centro Hospitalar Vila Nova de Gaia/Espinho, EPE
	Hospital Espírito Santo de Évora, EPE
	Hospital Fernando Fonseca, EPE
	Hospital Garcia de Orta, EPE
	Hospital de Braga, EPE
Group E	Centro Hospitalar Universitário Lisboa Norte, EPE
	Centro Hospitalar Universitário de Lisboa Central, EPE
	Centro Hospitalar Universitário de São João, EPE
	Centro Hospitalar Universitário do Porto, EPE
	Centro Hospitalar de Lisboa Ocidental, EPE
	Centro Hospitalar e Universitário de Coimbra, EPE

**Table 8:** The differences in performance for the top entities of groups B, C, and E, before and after the start of the pandemic

**Group B top entities**	**Average performance pre-March 2020**	**Average performance post-March 2020**	**Time considered benchmark pre-March 2020 (%)**	**Time considered benchmark post-March 2020 (%)**
Centro Hospitalar Póvoa de Varzim/Vila do Conde, EPE	0.984	0.990	59%	42%
Hospital Santa Maria Maior, EPE	0.977	0.977*	33%	23%
**Group C top entities**	**Average performance pre-March 2020**	**Average performance post-March 2020**	**Time considered benchmark pre-March 2020 (%)**	**Time considered benchmark post-March 2020 (%)**
Centro Hospitalar Entre Douro e Vouga, EPE	0.962	0.975	21%	19%
Hospital de Cascais, PPP	0.975	0.988	36%	54%
**Group D top entities**	**Average performance pre-March 2020**	**Average performance post-March 2020**	**Time considered benchmark pre-March 2020 (%)**	**Time considered benchmark post-March 2020 (%)**
Centro Hospitalar Vila Nova de Gaia/Espinho, EPE	0.980	0.982	18%	46%
Hospital de Braga	0.992	0.984	49%	62%

*The value is 0.9774, compared to 0.9767 prior to the pandemic. This entity also increased its performance although the value is the same in the table due to rounding

## Footnotes

1 INE. (2022). *Instituto Nacional de Estatística – Conta Satélite da Saúde.* Available at https://www.ine.pt/xportal/xmain?xpid=INE&xpgid=ine_destaques&DESTAQUESdest_boui=540875581&DESTAQUESmodo=2 accessed on 14/11/2022.

2 ACSS—*Administração Central do Sistema de Saúde.* Available at https://benchmarking-acss.min-saude.pt/ accessed on 18/12/2022.
